# MAJATNet: A Lightweight Multi-Scale Attention Joint Adaptive Adversarial Transfer Network for Bearing Unsupervised Cross-Domain Fault Diagnosis

**DOI:** 10.3390/e27101011

**Published:** 2025-09-26

**Authors:** Lin Song, Yanlin Zhao, Junjie He, Simin Wang, Boyang Zhong, Fei Wang

**Affiliations:** 1 School of Automobile and Transportation, Chengdu Technological University, Yibin 644012, China; 2 School of Electrical Engineering, Southwest Jiaotong University, Chengdu 610031, China; 3 School of Economics and Management, Panzhihua University, Panzhihua 617000, China

**Keywords:** bearings, multi-scale, attention mechanism, joint loss, transfer learning, fault diagnosis

## Abstract

Rolling bearings are essential for modern mechanical equipment and serve in various operational environments. This paper addresses the challenge of vibration data discrepancies in bearings across different operating conditions, which often results in inaccurate fault diagnosis. To tackle this related limitation, a novel lightweight multi-scale attention-based joint adaptive adversarial transfer network, termed MAJATNet, is developed. The proposed network integrates a feature extraction network innovation module with an improved loss function, namely IJA loss. The feature extraction module employs a one-dimensional multi-scale attention residual structure to derive characteristics from monitoring data of source and target domains. IJA loss evaluates the joint distribution discrepancy of high-dimensional features and labels between these domains. IJA loss integrates a joint maximum mean discrepancy (JMMD) loss with a domain adversarial learning loss, which directs the model’s focus toward categorical features while minimizing domain-specific features. The performance and advantages of MAJATNet are demonstrated through cross-domain fault diagnosis experiments using bearing datasets. Experimental results show that the proposed method can significantly improve the accuracy of cross-domain fault diagnosis for bearings.

## 1. Introduction

Rolling bearings are essential for modern mechanical equipment like CNC tools, wind turbines, and aircraft systems. Due to the rolling bearing taking a long time to load and transfer power [1], it is very susceptible to pitting, wear, spalling, scuffing, gluing, and other faults. If a bearing fault occurs, these faults can lead to periodic pulse shocks, jeopardizing equipment safety [2] and potentially causing significant damage or injuries [3,4]. Research shows that bearing failures or abnormalities account for about 40% of downtime in rotating machinery [5], emphasizing the need for timely and accurate fault detection [6,7].

The research achievements in fault diagnosis can be categorized into three methods: physical model-based methods, signal processing-based methods, and data-driven methods. Physical model-based methods typically simplify complex mechanical systems and have difficulty accurately diagnosing fault types. Signal processing-based methods can effectively carry out fault diagnosis by analyzing bearing monitoring signals, especially stochastic resonance technology, which is widely used for weak fault feature extraction under noisy data [8,9]. However, stochastic resonance methods usually require fine parameter tuning and have limited adaptability regarding complex distribution changes in multi-source domains. In addition, with the increasing complexity of mechanical equipment, physical models and signal-processing-based methods rely heavily on prior physical knowledge and manual experience, thus failing to meet the requirements of intelligent fault diagnosis [10].

Data-driven approaches are further categorized into shallow machine learning-based approaches and deep learning (DL)-based approaches [6]. In shallow machine learning models, methods such as K-Nearest Neighbors [11] and Naive Bayes [12,13,14] have developed rapidly. However, shallow machine learning methods require domain expertise to construct manual features, which hinders their industrial promotion and application. Recently, DL has become a feasible method for diagnosing bearing faults by modeling the complex relationship between easily accessible vibration signals and difficult-to-access bearing failure [15]. Unlike shallow machine learning, which relies on manual feature extraction, DL automates feature extraction by superimposing multilayer neural networks and nonlinear transformations, and has the following abilities: autonomous learning, automatic updating, and self-adaptation. The DL-based implementation approach reduces the need for human intervention and domain expertise [16]. For instance, to reduce the dependence on mechanical expertise, Guo et al. [17] proposed a bearing fault diagnosis method based on a deep convolutional neural network (CNN), which achieved satisfactory performance in both fault category and fault size identification; Wang et al. [18] proposed a fault diagnosis method based on compressed sensing and deep learning for the problem of limited data in real industrial scenarios, which successfully improved the fault diagnosis accuracy. Aiming at the problem of noise interference mixed in the signals collected by vibration sensors, Shi et al. [19] proposed an approach based on complete ensemble empirical model decomposition and improved the deep CNN, with an average fault diagnosis accuracy as high as 99.25%. Some emerging deep learning models, such as generative adversarial networks and Transformers, have brought new solutions for bearing fault diagnosis. For example, Zhou et al. [20] proposed a deep convolutional generative adversarial network for fault diagnosis under data constraints. Jin et al. [21] proposed a Transformer model capable of processing one-dimensional temporal data for rotating machinery fault diagnosis. Although these methods can improve the accuracy of bearing fault diagnosis, they do not take into account the adverse effects of changes in mechanical equipment operating conditions on data distribution, which limits their widespread application in industrial practice.

Despite the many advances in DL-based bearing fault diagnosis, the above methods are premised on the two assumptions that the training and test data must come from the same distribution and that there is sufficient labeled data for training [22]. In this context, there are typically two domains: the source domain for training and the target domain for testing the model performance [23]. However, in practical industrial applications of bearings, varying operational conditions can cause mismatched data distributions [24]. Additionally, labeled target domain samples will be even more difficult to collect, and sometimes impossible to make labels for this data. The monitoring data collected in practical applications usually do not satisfy the two assumptions, complicating DL-based unsupervised fault diagnosis [25].

Recently, domain adaptive technology, a type of transfer learning, has become a promising approach to deal with the above dilemma by applying knowledge gained from the source domain to the target domain [24]. Extensive literature reveals that current domain adaptation methods can be divided into two categories [26]. The first category involves mapping approaches where data from both domains is transformed into the Reproducing Kernel Hilbert Space (RKHS) to measure and minimize the distance between their distributions. This helps in extracting domain-invariant features. Common measures for this purpose include Max Mean Discrepancy (MMD), Multi-Kernel MMD (MK-MMD), and Correlation Alignment (CORAL). For instance, Sun et al. [27] proposed an MMD-based method for bearing unsupervised cross-domain fault diagnosis (UCFD), which is capable of measuring the marginal distribution distance between two domains. Xiao et al. [28] addressed the assumption that the consistency of data distribution between two domains is not satisfied due to frequent changes in the machine operating environment, and proposed a generalized metric matrix combined with MMD to improve the accuracy of cross-domain fault diagnosis. Guo et al. [29] employed the MMD distance to measure the discrepancy between the data generated by the diffusion model and the real data, applying it to fault diagnosis under unknown operating conditions. An et al. [30] developed a multi-layer, multi-kernel approach based on MK-MMD for rotating machinery failure detection. For the lack of labeled data for new working conditions, Che et al. [31] first pre-trained a deep belief network model from labeled samples consisting of the original vibration signals and their corresponding time and frequency domain metrics, and then applied the domain adaptive method in transfer learning to calculate the MK-MMD distance between the known condition data and the new condition data. To address the same problem, Shao et al. [32] utilized one-dimensional CNN and CORAL to assess distributional differences that successfully extract robust domain-invariant features. However, these methods only studied the marginal distribution differences in data between domains, without considering the impact of joint distribution differences.

The second is an adversarial learning-based approach [33]. This method treats the source domain data as real and the target domain data as generated, using adversarial training to extract domain-invariant characteristics. For example, to address the problem in a real industry where the two previous assumptions cannot be satisfied, Han et al. [34] integrated adversarial learning with CNNs to create a fault diagnosis framework with additional discriminative classifiers. More robust feature information can be extracted by adding additional discriminant networks. Liu et al. [35] developed an adversarial learning-based transfer learning for rolling bearing failure identification. The method performs feature extraction through stacking autoencoders and reduces the distributional differences in features in different domains through adversarial learning, which can effectively solve the problems of lacking labeled data and inconsistent data distribution. Chen et al. [36] introduced a transfer learning method that employs CNN and an encoder to learn features with hierarchical mechanisms and then reduces the distribution differences between the two domains through adversarial learning. However, adversarial learning-based methods suffer from unstable model training and are prone to inconsistencies between the feature space and the label space.

With the advancement of embedded devices, lightweight models have garnered widespread attention. Many existing deep learning models suffer from large parameter sizes and high computational complexity, failing to meet the industrial demand for lightweight and high-performance solutions [37]. Fang et al. [38] adopted a depthwise separable convolution to propose a lightweight fault diagnosis method for bearings. Zhong et al. [39] developed a fault diagnosis model for resource-constrained devices based on the SqueezeNet method and a self-attention mechanism. Wan et al. [40] combined the ShuffleNetV2 network with variational mode decomposition and fast Kurtogram algorithms for bearing fault diagnosis. Huang et al. [41] applied MobileNet to rotating machinery fault diagnosis using depthwise separable convolution and pointwise convolution. However, these methods do not extract multi-scale features from vibration data and fail to account for feature discrepancies caused by domain shifts. Yan et al. [42] constructed a lightweight fault diagnosis model using separable convolution and a Transformer, while Wu et al. [43] employed an improved multi-scale separable convolution and a Transformer to enhance both local and global feature representation. Nevertheless, these approaches also do not investigate the adverse impact of domain differences on the extraction of domain-invariant features.

According to reference [44,45,46,47], whether it is a mapping-based approach or an adversarial learning-based approach, the feature extraction backbone network based on one-dimensional convolutional operation can efficiently learn the category features from vibration signals, which exhibits excellent performance. However, the current domain adaptive technique still has some limitations: (1) Many assume different marginal distributions for the two domain data but overlook joint distribution discrepancies of high-dimensional features and labels, which reduces the prediction performance of the failure diagnosis models. (2) Mapping-based techniques adopt approximations that hinder precise measurement of joint distribution discrepancies between domains, making it challenging for a single domain adaptive technique to ensure a similarity of features across domains. (3) Current feature extraction networks struggle to capture both local and global multi-scale information effectively, with a large number of parameters and a lack of mechanisms to distinguish the importance of different scales. Treating all scales equally can lead to redundant feature transfer, complicating the extraction of domain-invariant characteristics. The model struggles to balance multi-scale feature extraction, parameter size, and diagnostic accuracy in practical applications.

To address current methods’ limitations, a novel lightweight multi-scale attention joint adaptive adversarial transfer network (MAJATNet) is developed. The first motivation is to develop a lightweight convolutional-based feature extraction backbone network that extracts crucial multi-scale features without adding extra parameters, improving the ability to capture discriminatory category-specific features. The second motivation is to propose a new domain adaptive loss function that is more effective at reducing joint distribution discrepancies between domains, facilitating the extraction of domain-invariant features. The core innovations include the following:

(1) A novel lightweight multi-scale attention residual network (MAResNet) is proposed, which utilizes multi-scale group convolution (MSGConv) to extract features at different scales and incorporates a one-dimensional efficient channel attention (1D-ECA) mechanism to dynamically recalibrate the importance of channel-wise features. This enhances the ability to capture discriminative fault-related features while significantly reducing model parameters and computational complexity.
(2) A novel loss function, namely IJA loss, is developed, which integrates JMMD and adversarial learning loss. The IJA loss function effectively measures the joint distribution discrepancy of high-dimensional features and labels between the source and target domains, facilitating the extraction of domain-invariant features and improving cross-domain fault diagnosis performance.
(3) MAResNet featurefeatures an extraction backbone and the IJA loss function areis integrated into a unified end-to-end adversarial transfer learning framework. This holistic approach jointly optimizes feature extraction, domain adaptation, and classification.

The structure of the paper is as follows: Section 2 introduces related work on UCFD. Section 3 introduces the proposed MAJATNet model in detail. Section 4 assesses the model’s performance and advantages. Section 5 offers concluding remarks.

## 2. Related Works

This part provides a brief description of the problem definition and MMD losses relevant to the topic of this paper.

### 2.1. UCFD Problem Definition

To better describe the UCFD of bearings, the problem is formulated [25].

(1) The source domain is defined as follows:

(1) $$D_{s}={\left\{\left(\left(x_{i}^{s},y_{i}^{s}\right)|\left(x_{i}^{s},y_{i}^{s}\right)\sim P(x^{s},y^{s})\right)\right\}}_{i=1}^{n_{s}}$$

where $y^{s}={\left\{y_{i}^{s}\right\}}_{i=1}^{n_{s}}$ is the label of the source domain input sample $x^{s}={\left\{x_{i}^{s}\right\}}_{i=1}^{n_{s}}$, the marginal probability distribution of $\left(x^{s},y^{s}\right)$ is $P\left(x^{s}\right)$, and the joint distribution of $\left(x^{s},y^{s}\right)$ is $P\left(x^{s},y^{s}\right)$.

(2) The target domain is defined as

(2) $$D_{t}={\left\{\left(\left(x_{j}^{t}\right)|\left(x_{j}^{t},y_{j}^{t}\right)\sim P(x^{t},y^{t})\right)\right\}}_{j=1}^{n_{t}}$$

where the target domain input sample $x^{t}={\left\{x_{j}^{t}\right\}}_{j=1}^{n_{t}}$ is unlabeled, the marginal probability distribution of $\left(x^{t},y^{t}\right)$ is $Q\left(x^{t}\right)$, and the joint distribution of $\left(x^{t},y^{t}\right)$ is $Q\left(x^{t},y^{t}\right)$.

(3) The marginal distribution and joint distribution do not coincide in the following:

(3) $$P\left(x^{s}\right)\neq Q\left(x^{t}\right)$$

(4) $$P\left(x^{s},y^{s}\right)\neq Q\left(x^{t},y^{t}\right)$$

### 2.2. Max Mean Discrepancy

To extract feature information that does not change with the domain, a loss function is needed to quantify data distribution discrepancies, aiming to minimize this loss during the training of the DL model. The MMD method evaluates the discrepancy by mapping high-dimensional features from both domains into an RKHS, which is a type of function. In the RKHS, each function is representable as a vector in the inner product space. The inner product in the RKHS defines a special kind of kernel function that has a reproduction property. By means of Gaussian kernel function, mapping a random variable to infinite dimensions and then solving for the expectation can yield arbitrary order moments of the random variable. The kernel RKHS function can be represented as follows [48]:

(5) $$k(x,x^{\prime })={\left\langle f(x),f(x^{\prime })\right\rangle }_{H}$$

where $k(x,x^{\prime })$ is the RKHS function denoted by the inner product $\langle \cdot ,\cdot \rangle_{H}$; H denotes the RKHS. The probability distribution P in the RKHS defined by the kernel $k(x,x^{\prime })$ can be expressed as follows:

(6) $$\mu_{x}(P)≜E[f(x)]=\int f(x)dP(x)$$

where $\mu_{x}(P)$ denotes the kernel mean embedding, and E denotes the mathematical expectation. The true probability distribution P is rarely available, but can be estimated using n finite samples, and in practice, the unbiased estimation of expectation is the mean. The estimate μ^x(P) can be represented by [49]:

(7) μ^x=1n∑i=1nf(xi)

The principle of MMD is that if the moments of any order of two random variables are the same, the two distributions are the same, and when the two distributions are not the same, the moment with the greatest distance gauges the discrepancy. In MMD-based failure recognition, source and target domain data are first processed through a CNN to extract features, which are then flattened, adopting a global average pooling (GAP) operation. Finally, the MMD loss is employed to align features from both domains and optimize model training. The MMD loss function [50] is given by the following:

(8) $$L_{MMD}(P,Q)=\underset{f\in H,∥f{∥}_{H}\leq 1}{sup}(E_{P}\left[f\left(x^{s}\right)\right]-E_{Q}\left[f\left(x^{t}\right)\right])$$

where $L_{MMD}(P,Q)$ denotes the MMD loss, sup denotes the upper boundary, f(·) indicates a mapping function, and ${∥f∥}_{H}\leq 1$ indicates that the Norm in the RKHS should be less than or equal to 1. Substituting Equation (7) into Equation (8) yields the following [25]:

(9) $$L_{MMD}(P,Q)=∥\frac{1}{n}\sum_{i=1}^{n}f(x_{i}^{s})-\frac{1}{m}\sum_{j=1}^{m}f(x_{j}^{t}){∥}_{H}$$

where n and m represent the sample capacity contained in the source and target domains, respectively. ${∥\cdot ∥}_{H}$ denotes the MMD distance. Furthermore, $L_{MMD}(P,Q)$ can be expressed as follows:

(10) $$\begin{array}{ll} L_{MMD}^{2}\left(P,Q\right) & =∥\frac{1}{n}\sum_{i=1}^{n}f\left(x_{i}^{s}\right)-\frac{1}{m}\sum_{j=1}^{m}f\left(x_{j}^{t}\right){∥}_{H}^{2}\\ & =∥\frac{1}{n^{2}}\sum_{i}^{n}\sum_{j}^{n}f(x_{i}^{s})f(x_{j}^{s})-\frac{2}{nm}\sum_{i}^{n}\sum_{j}^{m}f(x_{i}^{s})f(x_{j}^{t})+\frac{1}{m^{2}}\sum_{i}^{m}\sum_{j}^{m}f(x_{i}^{t})f(x_{j}^{t}){∥}_{H} \end{array}$$

## 3. Proposed MAJATNet Method

The MAJATNet method mainly includes MAResNet and the IJA loss function.

### 3.1. Multi-Scale Group Convolutional

**Figure 1:**
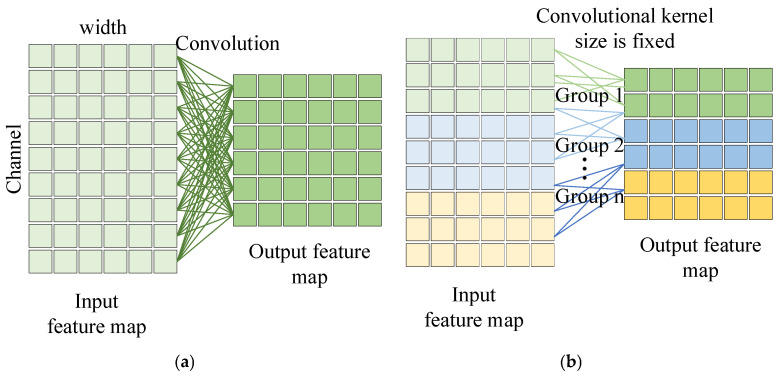
Different convolutional methods. (**a**) Ordinary convolutional; (**b**) Group convolutional.

Figure 1a,b represent the ordinary convolutional and group convolutional operations, respectively. In ordinary convolutional operations, each convolutional kernel is computed with the input to produce the corresponding output. Further inspired by the idea of sparse concatenation, group convolution first divides the input channel into different groups, and each group employs a different convolutional kernel to compute with it to produce corresponding sub-outputs, which are finally concatenated together as the final output. Group convolution can further decrease the parameters. The idea of group convolution is further used in the classical ResNeXt [51] model. However, in group convolution, different convolutional kernels are of equal size, which is not favorable for the model to extract multi-scale features. Generally, large convolutional kernels can extract global information, and small convolutional kernels are able to capture detailed information.

**Figure 2:**
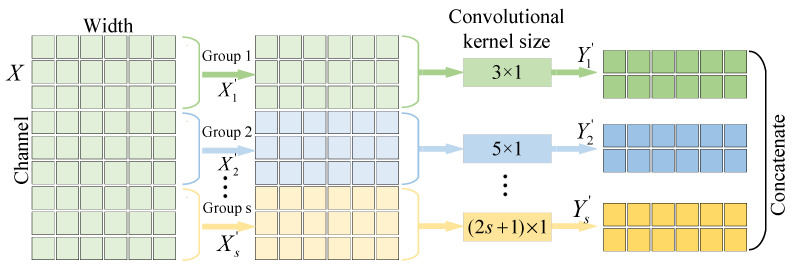
Schematic diagram of MSGConv.

Based on group convolution, a new one-dimensional MSGConv structure for UCFD of bearings is proposed, as shown in Figure 2. In the input data where $X\in R^{C\times 1\times W}$, W and C represent the width and channel, respectively. MSGConv first splits the input data into different groups along the channel, $X^{\prime }=\left[X_{1}^{\prime },X_{2}^{\prime },X_{3}^{\prime },\ldots \ldots {,X}_{s}^{\prime }\right]$, where $X_{i}^{\prime }$ represents the i-th group data. $X_{i}^{\prime }\in R^{(C/s)\times 1\times W}$, where s represents the total number of groups. Then, a convolutional operation is performed for each group, where different groups adopt different convolutional kernel sizes for extracting multi-scale feature information. The output $Y_{i}^{\prime }$ corresponding to $X_{i}^{\prime }$ is ${Conv}_{i}(X_{i}^{\prime })$. The convolutional kernel size of ${Conv}_{i}(\cdot )$ is represented as (2i+1)×1. Finally, the extracted multi-scale feature information is concatenated together along the channel dimensions to achieve the output $Y^{\prime }=\left[Y_{1}^{\prime },Y_{2}^{\prime },Y_{3}^{\prime },\ldots \ldots {,Y}_{s}^{\prime }\right]$.

In addition, it should be especially noted that this one-dimensional MSGConv is a lightweight model, assuming that the input channel, output channel, and kernel size of the convolutional layer are $C_{1}$, $C_{2}$ and $k_{1}\times 1$, respectively. The parameters $P_{OC}$ of the ordinary convolution is represented as follows:

(11) $$P_{OC}=k_{1}C_{1}C_{2}$$

The first convolutional kernel size of MSGConv is $k_{1}\times 1$, and there are s groups in total. The convolutional kernel size of MSGConv generally presents an equidistant series with a tolerance of 2. Then the number of parameters $P_{MGC}$ of MSGConv is as follows:

(12) $$P_{MGC}=\frac{C_{1}C_{2}(k_{1}+2s+1)}{2s}=C_{1}C_{2}(\frac{\left(k_{1}+1\right)}{2s}+1)$$

Typically, $k_{1}\geq 3$; s≥2. It can be shown that $P_{MGC}\gg P_{OC}$. The MSGConv structure not only extracts multi-scale features, but also greatly decreases parameters. This advantage helps to alleviate the problems of high hardware overhead and difficult parameter optimization in DL.

### 3.2. Multi-Scale Attention Residual Network

**Figure 3:**
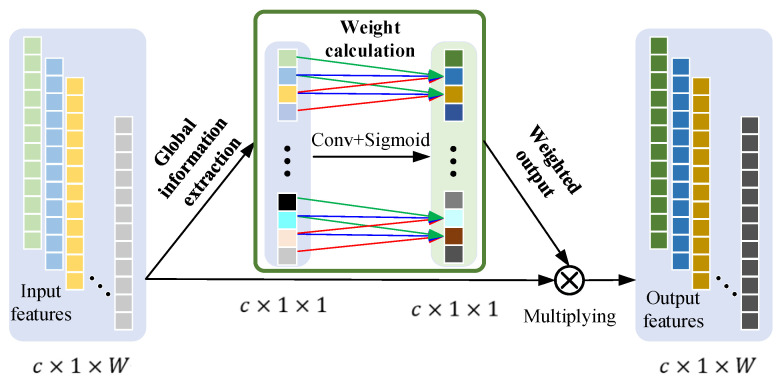
1D-ECA attention mechanism.

Each group of features obtained by MSGConv contains different scale information. Different faults may contain features at different scales. Some of the early faults have weak impulse signals, and the feature information may be distributed on a large scale. Severe bearing failures may result in large impulses with significant features at a small scale. Therefore, various failure types and various stages of the identical failure can be characterized at different scales. Some scales of information extracted by MSGConv may contain a large number of features related to bearing faults, while at some other scales, they may contain few fault-related features or predominantly noise interference information. To distinguish discriminative and significant feature representation, modeling the inter-channel relationships along the channel dimensions and thus recalibrating the importance of different scales information is essential, which contributes to the enhancement of the feature learning capability of DL networks. This paper adopts 1D-ECA [52] to dynamically adjust the importance of channel information, enabling the model to emphasize data relevant to bearing failures while suppressing redundant information. As shown in Figure 3, 1D-ECA mainly includes three steps: global information extraction, weight calculation, and weighted output.

Global information extraction: To obtain the relationship between different channels, global spatial information needs to be compressed into the channel descriptions, which is achieved by a GAP operation. The global description of statistical information z in the c-th channel can be expressed as follows:

(13) $$z_{c}=\frac{1}{W}\sum_{j=1}^{W}x_{c}(1,j)$$

where $x\in R^{c\times 1\times W}$ is a one-dimensional vibration signal. $x_{c}(1,j)$ represents the data in channel c, row 1, and column j. $z\in R^{c\times 1\times 1}$, $z_{c}$ is the channel statistic that captures the global information along the channel dimension.

Weight calculation: To obtain the intrinsic correlations among channels, this step requires the ability to learn nonlinear interactions between different channels. This is accomplished using a 1D-CNN layer Conv and a Sigmoid activation function. The output channels and kernel size of 1D-CNN are 1 and 3×1, respectively. The arithmetic mechanism of the Conv is able to establish the interactions between the channels. The Sigmoid outputs values are between 0 and 1, which ensure that more than one channel is emphasized. The obtained attention weight a can be expressed as follows:

(14) a=Sigmoid(Conv(z))

Weighted output: Multiplying the input features x and the attention weights obtain a weighted output. The adaptively recalibrated output ${\bar{x}}_{c}$ can be expressed as follows:

(15) $${\bar{x}}_{c}=a_{c}x_{c}$$

**Figure 4:**
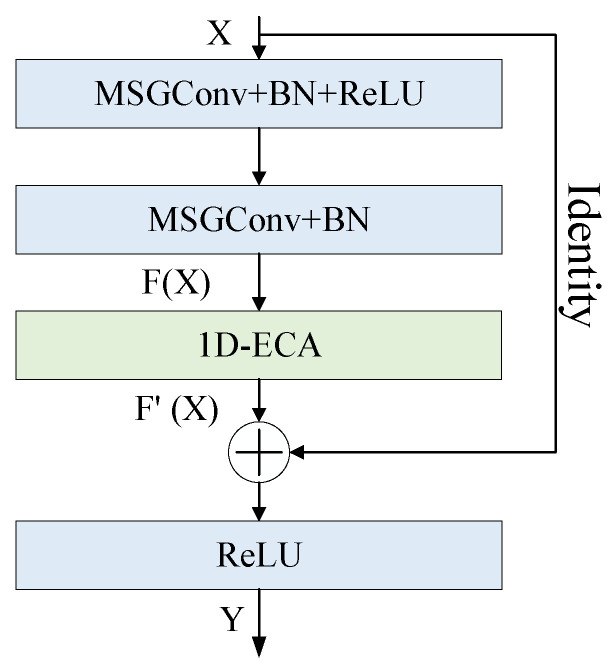
The residual block of MAResNet.

**Figure 5:**
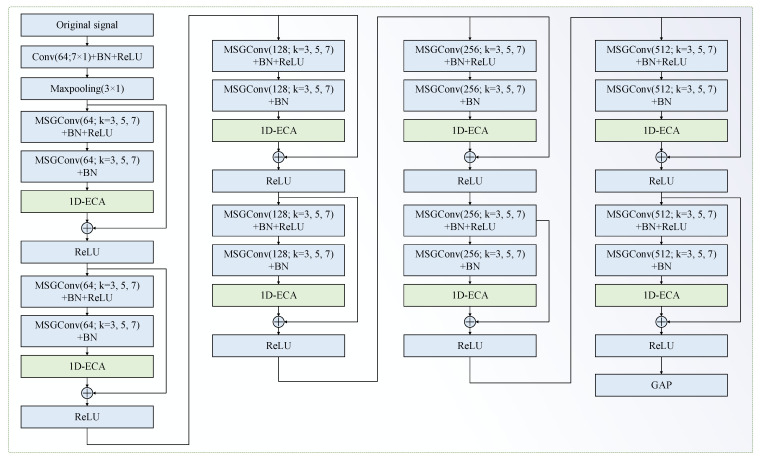
The developed MAResNet.

To improve the model’s capability of extracting multi-scale characteristics and to enhance the performance of UCFD for bearings, an improved residual network, namely MAResNet, is constructed by adding MSGConv and the 1D-ECA mechanism module into the residual network. The residual block of MAResNet contains two independent paths, as shown in Figure 4. Path 1 is the MSGConv layer → Batch Normalization (BN) layer → ReLU activation function layer → MSGConv layer → BN layer → 1D-ECA mechanism layer, and path 2 is an identity. As illustrated in Figure 5, MAResNet is adopted as the feature extraction backbone network. Conv(64;7×1) means an output channel number of the convolutional layer is 64; the kernel size is 7×1. Maxpooling (3 × 1) indicates that the size of the pooling region is 3×1. The number of Conv and the Maxpooling sliding stride is 2. MSGConv(64;k=3,5,7) means an output channel number of the multi-scale convolutional operation is 64; the convolution is divided into three groups with convolutional kernel sizes of 3×1, 5×1 and 7×1. The convolutional kernel sliding stride is 1.

### 3.3. IJA Loss Function of MAJATNet

Accurately measuring the joint distribution distance between two domains is challenging. The approximations employed by mapping-based techniques hinder the precise quantification of the discrepancies in joint distributions across domains. Adversarial-based methods suffer from unstable model training and are prone to inconsistencies between the feature space and the label space. Therefore, neither mapping-based nor adversarial-based approaches alone can guarantee the extraction of stable domain-invariant features. Single optimization methods have inherent limitations and struggle to adapt to diverse datasets. The core idea of the IJA loss is to combine the advantages of both methodologies, addressing the problem from different dimensions to encourage the model to extract fault category features that are as immune as possible to domain shifts. Specifically, during model training, it is necessary to simultaneously optimize the JMMD loss, the domain adversarial loss, and the cross-entropy classification loss. Optimizing the JMMD and adversarial losses promotes parameter updates that enhance feature invariance to domain variations, while optimizing the classification loss drives the model to increase inter-class discriminability of input samples.

(1) JMMD loss

MMD has been widely utilized to calculate the marginal distribution distance between two domains. However, MMD cannot address the joint distribution discrepancy problem of $P\left(x^{s},y^{s}\right)\neq Q\left(x^{t},y^{t}\right)$. Transfer learning is more challenging when the discrepancy of the joint distribution leads to a change in the learning domain. This is a common scenario in practical applications. The transferability of features and classifiers is further degraded when the domain discrepancy is large. The original joint distribution is estimated by inference using the joint distribution of activations in a particular neural network layer. The kernel mean embedding is generalized to the joint distribution by means of the second-order tensor product feature space $H_{1}⊗H_{2}$:

(16) $$\mu_{x,y}\left(P\right)≜E_{x,y}\left[f_{H_{1}}\left(x\right)⊗f_{H_{2}}\left(y\right)\right]=\int f_{H_{1}}\left(x\right)⊗f_{H_{2}}\left(y\right)dP(x,y)$$

where ⊗ denotes the outer product, $f_{H_{1}}\left(x\right)$ denotes the feature mapping function for the variable x in the RKHS, and $f_{H_{2}}\left(y\right)$ is the mapping function for the variable y. The true probability distribution P is rarely available but can be estimated using n finite samples. The estimate μ^x,y(P) of $\mu_{x,y}\left(P\right)$ is given as follows:

(17) μ^x,y=1n∑i=1nfH1x⊗fH2y

The JMMD, based on joint distribution, integrates the high-dimensional representation and label. Unlike Wasserstein distance or MMD, which only calculate the distance between high-dimensional features, JMMD unifies high-dimensional features and classifier training into a single objective function for optimization. This enables the feature extractor to learn features that are not only domain-invariant but also highly discriminative for the classification task, thereby achieving more intrinsic domain alignment. Noted that since target domain samples’ labels do not exist, the label classification module’s predicted outputs (pseudo-labels) are used as labels for samples in the target domain during JMMD calculation. While early model predictions may be incorrect, most samples are accurately labeled as the network parameters are updated, complying with UCFD regulations. Thus, the JMMD loss can be expressed as

(18) LJMMD(P,Q)=∥μ^xs,ys−μ^xt,yt∥H1⊗H2

where ${∥\cdot ∥}_{H_{1}⊗H_{2}}$ denotes the JMMD distance in the second-order tensor product feature space $H_{1}⊗H_{2}$; $L_{JMMD}$ denotes the JMMD loss. Further generalization of the kernel function k is as follows:

(19) $$L_{JMMD}^{2}\left(P,Q\right)=∥\frac{1}{n^{2}}\sum_{i}^{n}\sum_{j}^{n}k_{H_{1}}\left(x_{i}^{s},x_{j}^{s}\right)k_{H_{2}}\left(y_{i}^{s},y_{j}^{s}\right)\;-\frac{2}{nm}\sum_{i}^{n}\sum_{j}^{m}k_{H_{1}}\left(x_{i}^{s},x_{j}^{t}\right)k_{H_{2}}\left(y_{i}^{s},y_{j}^{t}\right)+\frac{1}{m^{2}}\sum_{i}^{m}\sum_{j}^{m}k_{H_{1}}(x_{i}^{t},x_{j}^{t})k_{H_{2}}(y_{i}^{t},y_{j}^{t})∥$$

(2) Domain adversarial loss

**Figure 6:**
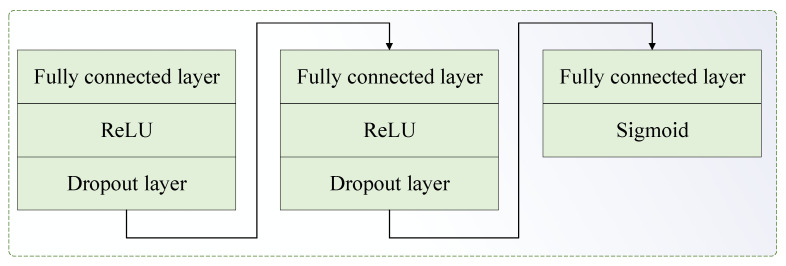
Domain discriminator network structure.

**Figure 7:**
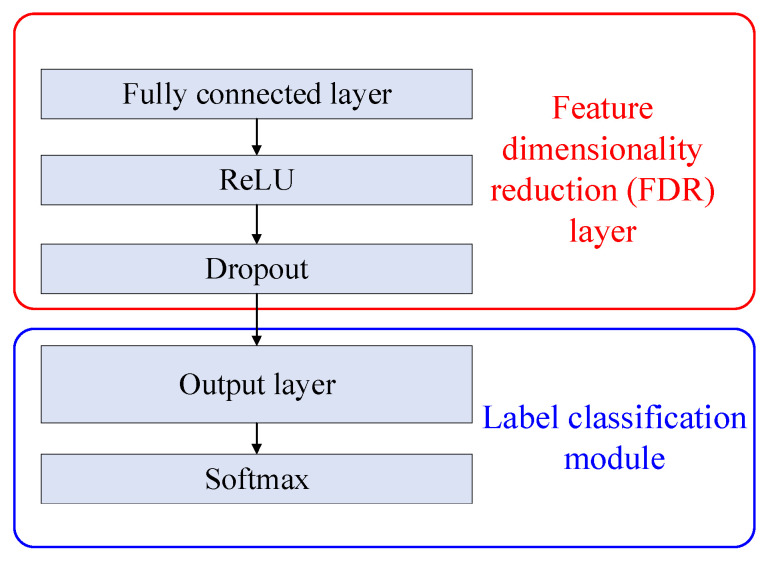
FDR layer and the label classification module.

Following the adversarial learning approach [33], the source and target domain data are treated as real and generated data, respectively. In this setup, the generator’s role shifts from generating data to functioning as a deep characteristic extraction network. The generator aims to extract domain-invariant features that remain consistent across domains, making it difficult for the discriminator to identify the source of the data [53]. The discriminator’s role is to determine whether these features originate from the source or target domain. By adversarially training the feature extractor and discriminator, the target domain feature distribution is expected to fit the source domain features as closely as possible. The developed domain discriminator contains the following network layers: three fully connecteds, two ReLUs, two dropouts, and a Sigmoid, as presented in Figure 6. In the same manner as JMMD loss considers joint distribution discrepancy, joint distribution influence is also considered in adversarial learning loss. The input data of the domain discriminator model consists of high-level feature output from the feature dimensionality reduction (FDR) layer and predicted label output from the label classification module. The FDR layer is shown in Figure 7. During adversarial training, $x_{i}$ represents the data, the loss L for each sample adopts a negative log-likelihood loss, which is given as

(20) $$L(G_{d}(G_{f}(x_{i})),d_{i})=d_{i}log(G_{d}(G_{f}(x_{i})))-(1-d_{i})log(1-G_{d}(G_{f}(x_{i})))$$

where $d_{i}$ is the domain label; $d_{i}$ is 1 if the i-th data is from the source domain and 0 if it is from the target domain. $G_{d}$ is the binary classification discriminator. $G_{f}$ is the feature extractor. The domain adversarial loss s given as

(21) $$L_{d}=-E_{x_{i}^{s}\in D_{s}}\log\left[G_{d}\left(G_{f}\left(x_{i}^{s}\right)⊗G_{c}\left(G_{f}\left(x_{i}^{s}\right)\right)\right)\right]\;-E_{x_{i}^{t}\in D_{t}}log[1-G_{d}(G_{f}(x_{i}^{t})⊗G_{c}(G_{f}(x_{i}^{t})))]$$

where $G_{c}$ is the label classification module.

(3) Classification loss

The FDR layer and label classification module are structured as shown in Figure 7, where the data passes through the fully connected layer → ReLU → Dropout → Output → Softmax. The GAP layer in Figure 5 is connected to the FDR layer. The Softmax layer’s output is the probability of each category. The red box shows the FDR layer, and the blue box shows the label classification module.

The label classification module aims to minimize the cross-entropy loss of the source domain data, which is written as

(22) $$L(G_{c}(G_{f}(x_{i}));y_{i})=-\sum_{j=0}^{m-1}I(y_{i}=j)log(G_{c}(G_{f}(x_{i}))$$

where m stands for quantity of fault categories; I(·) is a function, and I=1 if $y_{i}=j$ and I=0 if $y_{i}\neq j$. The classification loss on the source domain data can be expressed as follows:

(23) $$L_{CEL}=\frac{1}{n_{s}}\sum_{i=1}^{n_{s}}L(G_{c}(G_{f}(x_{i}^{s}));y_{i}^{s})$$

(4) Overall objective functions

**Figure 8:**
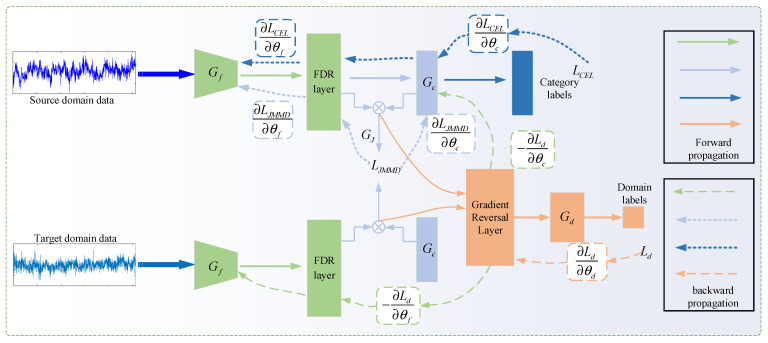
Overall framework of the MAJATNet model.

Accurately measuring the joint distribution’s distance between two domains is challenging due to the fact that a single loss function between domains may lose some feature information, and ensuring feature sufficiently similar across domains is difficult. To solve this problem and enrich the inter-domain loss function, this paper proposes the IJA loss, which combines cross-entropy loss, JMMD, and adversarial learning. The MAJATNet model includes four components: the backbone network module $G_{f}$, the domain discriminator module $G_{d}$, the label classification module $G_{c}$, and the JMMD module $G_{J}$ as shown in Figure 8. $G_{f}$ is the MAResNet developed in Section 3.2 for extracting high-dimensional features from data of two domains. The high-dimensional characteristics obtained by the MAResNet connecting the FDR layers are $FDR(G_{f}(x;\theta_{f}))$, where $\theta_{f}$ is the network parameter connecting $G_{f}$ and the FDR layer together. For simplicity, these features are denoted as $G_{f}(x;\theta_{f})$. These high-level characteristics are passed to the label classification module to obtain the corresponding output $G_{c}(G_{f}(x;\theta_{f});\theta_{c})$, where $\theta_{c}$ is the network parameter of $G_{c}$. Meanwhile, these features and labels are jointly used by the domain discriminator $G_{d}$ to determine their domain origin, and the output is $G_{d}({(G}_{f}\left(x;\theta_{f}\right),G_{c}(G_{f};\theta_{c}));\theta_{d})$, where $\theta_{d}$ is the network parameter of $G_{d}$. The JMMD algorithm, implemented in $G_{J}$, gauges the distance between two domains, producing output $G_{J}(G_{f}(x;\theta_{f}),G_{c}(G_{f};\theta_{c}))$.

As mentioned earlier, the IJA loss consists of three parts: cross-entropy classification loss, domain distance loss JMMD, and domain adversarial loss. During training, three losses are computed simultaneously to optimize the objective function and obtain category classification features and domain-invariant features. Parameters of $G_{d}$ need to be adjusted to minimize the $L_{d}$, enhancing the discriminator’s ability to differentiate the origin of data. Parameters of $G_{f}$ and $G_{c}$ are adjusted to reduce the $L_{CEL}$ loss so that the input sample categories are distinguishable. Parameters of $G_{f}$ and $G_{c}$ are optimized to minimize the $L_{JMMD}$ loss and maximize the $L_{d}$ loss so that the high-dimensional characteristics of two domains are indistinguishable, i.e., domain-invariant features are extracted. The overall objective loss $L_{IJA}$ of the MAJATNet model is

(24) $$L_{IJA}(\theta_{f},\theta_{c},\theta_{d})=L_{CEL}+\lambda_{1}L_{JMMD}-\lambda_{2}L_{d}$$

where $\lambda_{1}$ and $\lambda_{2}$ are the weight parameters for balancing the three losses. The MAJATNet model is trained to find the following optimal network parameters θ^={θ^f,θ^c,θ^d}:

(25) (θ^f,θ^c)=arg  minθf,θcLIJA(θf,θc,θ^d)

(26) (θ^d)=arg  maxθdLIJA(θ^f,θ^c,θd)

The parameter update can be expressed as follows:

(27) $$\theta_{f}\leftarrow \theta_{f}-\eta (\frac{\partial L_{CEL}}{\partial \theta_{f}}+\lambda_{1}\frac{\partial L_{JMMD}}{\partial \theta_{f}}-\lambda_{2}\frac{\partial L_{d}}{\partial \theta_{f}})$$

(28) $$\theta_{c}\leftarrow \theta_{c}-\eta (\frac{\partial L_{CEL}}{\partial \theta_{c}}+\lambda_{1}\frac{\partial L_{JMMD}}{\partial \theta_{c}}-\lambda_{2}\frac{\partial L_{d}}{\partial \theta_{c}})$$

(29) $$\theta_{d}\leftarrow \theta_{d}-\eta \lambda_{2}\frac{\partial L_{d}}{\partial \theta_{d}}$$

### 3.4. Training the MAJATNet Model

The feature extraction module $G_{f}$ is unstable in the early stage due to random initialization of parameters. To address this before undertaking the UCFD task, as illustrated in Algorithm 1, the model is first trained on labeled source domain data for 50 epochs to obtain a source domain pre-trained model and enhance the performance of the label categorization module. During the period, only calculate the $L_{CEL}$ loss. The model obtained excellent classification performance after 50 epochs of training, at which point cross-domain transfer learning was performed. The training data of the two domains are input into the model to calculate the $L_{CEL}$, $L_{JMMD}$, and $L_{d}$ loss, respectively, to minimize the overall loss and optimize the network parameters $\theta =\{\theta_{f},\theta_{c},\theta_{d}\}$. The trained model weights, biases, and model structure are saved as a local file when the maximum training epoch is reached, which is used for testing and deployment.
**Algorithm 1:** Training the MAJATNet model**Input:** source domain data $D_{s}={\left\{\left(x_{i}^{s},y_{i}^{s}\right)\right\}}_{i=1}^{n_{s}}$; target domain data $D_{t}={\left\{x_{j}^{t}\right\}}_{j=1}^{n_{t}}$; maximum epoch number E and pre-training epoch number $E_{p}$. 1: Initialize the parameters of the MAJATNet model $\theta =\{\theta_{f},\theta_{c},\theta_{d}\}$ 2: **for** epoch=1,2,3,……, E **do** 3:       **if**
$epoch\leq E_{p}$ **do** 4:         // Source domain data for pre-training 5:         // Forward propagation 6:      Compute the output of the feature extractor $G_{f}(x^{s};\theta_{f})$ and the output of the classifier $G_{c}(G_{f}(x^{s};\theta_{f});\theta_{c})$ 7:        Calculate the cross-entropy loss $L_{CEL}$ by Equation (23) 8:         // Backward propagation 9:         Updating the model parameters $\theta_{f}$ and $\theta_{c}$ by $\theta_{f}\leftarrow \theta_{f}-\eta \frac{\partial L_{CEL}}{\partial \theta_{f}}$ and $\theta_{c}\leftarrow \theta_{c}-\eta \frac{\partial L_{CEL}}{\partial \theta_{c}}$ 10:      **end if** 11:      **if**
$E_{p}<epoch\leq E$ **do** 12:          // Source and target domain data training 13:          // Forward propagation 14:      Compute the output of the feature extractor $G_{f}(x;\theta_{f})$, the output of the classifier $G_{c}(G_{f}(x;\theta_{f});\theta_{c})$ and the output of the discriminator $G_{d}({(G}_{f}\left(x;\theta_{f}\right),G_{c}(G_{f};\theta_{c}));\theta_{d})$ 15:    Calculate the cross-entropy loss $L_{CEL}$ by Equation (23), the JMMD loss $L_{JMMD}$ by Equation (19), and the adversarial learning loss $L_{d}$ by Equation (21) 16:      Calculate the overall objective loss $L_{IJA}$ by Equation (24) 17:          // Backward propagation 18:      Update the parameter $\theta_{f}$ by Equation (27), the parameter $\theta_{c}$ by Equation (28), and the parameter $\theta_{d}$ by Equation (29) 19:      **end if** 20: **end for** **Output:**
θ^={θ^f,θ^c,θ^d}


## 4. Experimental Verification

To assess the performance and advantages of the developed MAJATNet model for UCFD, this section includes experiments with two different bearing case studies.

### 4.1. JNU Dataset Experimental

(1) **Experimental platform and data processing**

**Figure 9:**
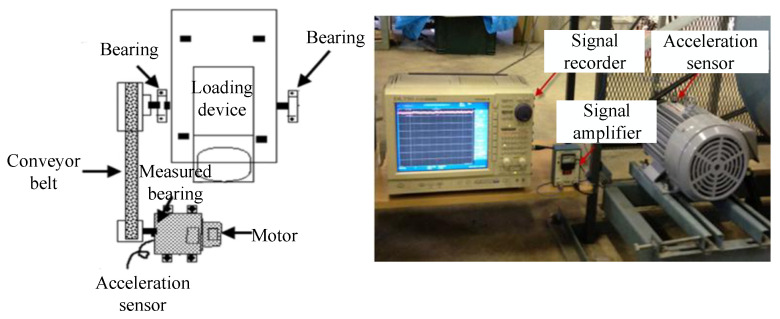
JNU fault diagnosis experimental platform.

**Figure 10:**
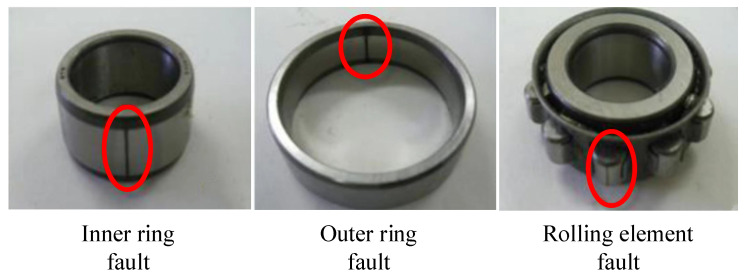
Three kinds of bearing failures.

The Jiangnan University (JNU) bearing experimental platform [54] is shown in Figure 9, which consists of a loading device, a conveyor belt, an induction motor, a bearing device, and a measurement device. The acceleration sensor (PCB MA352A60) obtains vibration data in the vertical direction with a sampling frequency of 50 kHz. The vibration signals collected by the acceleration sensor were amplified by a sensor signal amplifier (PCB ICP 480C02) and converted into a signal recorder. The test bench was run at 600 r/min, 800 r/min, and 1000 r/min speeds to simulate different working conditions. The bearing types are outer ring separable N205 and inner ring separable NU205, the outer diameter of the two bearings is 52 mm, the inner diameter is 25 mm, and the width is 15 mm; detailed parameters of the bearings are given in reference [54]. As shown in Figure 10, the dataset includes four health states: normal, inner ring fault, outer ring fault, and rolling element fault. The outer ring separable N205 was utilized for normal, outer ring, and rolling element fault data collection, and the inner ring separable NU205 was utilized for inner ring fault data collection. Bearing failures are artificially created by wire cutters.

Bearing data at three rotational speeds were utilized for the experimental evaluation, which contained a total of 12 sub-datasets, and 1024 data points were intercepted in each sub-dataset to form a sample. In each operating condition, 1466 normal samples and 1464 failure samples (488 failures in each category) are included. To assess the UCFD performance of the developed method when transferring from one domain to another, a total of six transfer tasks were developed. 0, 1, and 2 represent bearings serving in three different operating speeds. Different operating conditions are utilized to simulate data from different domains. For instance, in the first transfer task 0 → 1, and 0 represents the source domain data that was collected at 600 r/min; 1 represents the target domain data that was collected at 800 r/min. In each transfer experiment, the data from two domains are split into training and test sets with an 8:2 ratio.

(2) **Comparison methods and parameter settings**

The model’s performance evaluation index is accuracy, and the corresponding calculation formula can be expressed as

(30) $$Acc=\frac{Sum({Test}_{i}=={True}_{i})}{Sum({True}_{i})}\times 100\%$$

where Acc represents classification accuracy; $Sum({Test}_{i}=={True}_{i})$ represents predicted label size equal to true label. $Sum({True}_{i})$ indicates test sample size. To evaluate the performance and advantages of the MAJATNet model for UCFD, the developed model is compared with some advanced models. The methods for comparison are as follows.

**CNN**: The method ignores the distributional differences between two domains and does not employ domain adaptation techniques. **ResNet** [55]: Same as the CNN model, the ResNet model also ignores the problem of domain discrepancy. **AdaBN** [56]: BN helps address the internal covariance shift by updating parameters solely during training. It uses statistics information from the training data to normalize test samples. However, if the data distribution shifts, these statistics may not be applicable. AdaBN addresses this by substituting the statistics of each BN layer with information from the target domain during testing. **MK-MMD** [30]: MK-MMD is a mapping-based transfer learning method, which mainly includes two components: feature extraction and label classification. The data from two domains are feature extracted and then calculate MK-MMD and cross-entropy loss. **DA** [34]: The domain adversarial (DA) neural network mainly includes three components: feature extraction, label classification and adversarial learning. The data from two domains are feature extracted and then calculate adversarial learning and cross-entropy loss. **CDA** [25]: The difference between the conditional domain adversarial (CDA) method and the DA method is that the CDA method combines the high-dimensional characteristics and the predictive labels to form a joint distribution, which is then used for adversarial learning. **ShuffeResNet**: ShuffeNet [57] improves the predictive ability of lightweight models through group convolution and channel shuffling. The ShuffeResNet model is constructed by adding group convolutional and channel shuffling operations to ResNet, and the convolutional channels are divided into four groups. Comparative methods also include three lightweight models: SqueezeNet [39,58], LiConvFormer [42] (which combines convolution and Transformer methods), and MobileNetV2 [59]. Additionally, CSAN [60], LMMD [61] and JCSDAN [62], which employ state-of-the-art domain adaptation techniques, are used for comparison to validate the superiority of the proposed method.

Among the thirteen comparison methods, the CNN and ResNet are the classical DL models, which are employed as baseline models for comparing the feature extraction ability of DL models when no domain adaptation technique is utilized. AdaBN has been applied to transfer learning problems. MK-MMD is a mapping-based approach that contributes to minimizing the distributional differences between the extracted features from two domains and is the main domain adaptive technique. DA and CDA methods adopt adversarial learning to extract the shared features from two domains and are widely used in domain adaptation. ShuffeResNet, SqueezeNet, LiConvFormer, and MobileNetV2 are lightweight models that also employed the IJA loss during training; CSAN, LMMD, and JCSDAN are domain adaptation techniques, and the experimental results for these three methods are sourced from reference [62]. The above methods are compared with the proposed method in different aspects.

During training, The MAJATNet model and the comparative models, excluding CSAN, LMMD, and JCSDAN, utilize the same set of hyperparameters, as shown in Table 1. These hyperparameters followed the open-source settings in the benchmarking research literature [25]. In addition, the weighting coefficient $\lambda_{1}$ of the JMMD loss $L_{JMMD}$ increases the trade-off parameter from 0 to 1, employing an asymptotic training method [53], and the value of $\lambda_{1}$ can be expresse as follows:

(31) $$\lambda_{1}=\frac{1-e^{-(10k)}}{1+e^{-(10k)}}$$

where k∈[0,1] represents the training process, which is the current training epoch divided by the maximum training epoch after the activation of the transfer learning strategy. The trade-off parameter $\lambda_{2}$ for the adversarial learning loss $L_{d}$ has a value of 1. The experiments were conducted on RTX 2060 SUPER GPU utilizing the Pytorch 2.2.2 framework based on Python 3.11.

(3) **Comparative experimental results**

Five experiments were conducted for each model, excluding CSAN, LMMD, and JCSDAN, to reduce the interference of randomly initialized DL parameters on the results, and Table 2 illustrates the average test accuracy.

First, comparing the model parameters, the CNN has extremely low prediction accuracy in the six transfer learning tasks, although it has very low model complexity, parameters, and inference time. The ResNet, AdaBN, MK-MMD, DA, and CDA methods adopt the ordinary convolutional structure as the base module of the feature extraction backbone network, which has large computational complexity and parameters, in which the parameter of $G_{f}$ is 3.84 M. Recomputing the target domain’s statistical features in the inference phase leads to the longest inference time for AdaBN. Although the four excellent lightweight models—ShuffleResNet, SqueezeNet, LiConvFormer, and MobileNetV2—have fewer parameters, lower computational complexity, and shorter inference times compared to MAJATNet, their diagnostic accuracy is significantly lower than that of the MAJATNet model. MAJATNet significantly outperforms these lightweight models across all tasks. For example, in task 1 → 0, MAJATNet achieved 97.95%, which is markedly higher than ShuffleResNet’s 96.18%, SqueezeNet’s 93.31%, LiConvFormer’s 93.79%, and MobileNetV2’s 93.28%. In task 2 → 0, MAJATNet’s 94.98% also surpasses other models, with the next highest being ShuffleResNet’s 93.86%. Especially compared to the LiConvFormer model, there is a significant improvement, which indirectly reflects that although the Transformer model can achieve good prediction accuracy in the case of data with the same distribution, it is difficult for the Transformer model to cope with this dilemma when the domain shifts and the test data distribution diverges from that of the training data. Although some lightweight models perform closely in certain individual tasks, MAJATNet demonstrates significant advantages in both overall accuracy and stability. This indicates that while these lightweight models achieve extremely low parameter counts and model complexity, it comes at the cost of predictive accuracy. MAJATNet employs a group multi-scale convolutional structure with a moderate number of parameters and complexity. The parameter count of its $G_{f}$ module is 2.21 M, which is 42.2% fewer compared to the original convolutional structure. Additionally, the inference time for 586 test samples is 0.28 s (approximately 0.48 ms per sample), and while not the lowest, it remains well within an industrially acceptable range. MAJATNet is able to reduce parameters while improving predictive performance, achieving a better balance among parameter quantity, complexity, and prediction accuracy.

Table 2 demonstrates that the accuracies of the traditional DL models CNN, ResNet, and AdaBN in the six transfer learning tasks are relatively poor, especially the more challenging tasks such as 1 → 0 and 2 → 0. The accuracies for task 1 → 0 are 82.97%, 83.93% and 88.47%, and the accuracies for task 2 → 0 are 90.34%, 87.95% and 90.30%. Compared with MK-MMD, DA, and CDA methods, which employ domain adaptive techniques, the domain adaptive methods can achieve higher classification accuracy. This phenomenon indicates that although the deep convolutional models have a strong feature extraction capability to capture discriminative high-dimensional characteristics, the extracted characteristics are not domain adaptive. When the features learned in the source domain are generalized to the target domain, large data distribution differences between the two domains result in unsatisfactory prediction performance. The application of a domain adaptive technique better eliminates the distribution discrepancy and thus enhances the diagnosis capability of the model.

Table 2 also reveals that the MAJATNet model has higher accuracies, all greater than 94.9%, in the six transfer tasks, and smaller standard deviations on most of the tasks. Compared to the MK-MMD method, the accuracies of six transfer tasks were improved. In the tasks 0 → 2, 1 → 0, and 2 → 0, which are more challenging to categorize with MK-MMD, MAJATNet accuracy improved by 0.99%, 1.02% and 1.39%, respectively. Compared to the DA method, the accuracies of six transfer tasks were improved. In the transfer tasks 0 → 2, 1 → 0, and 2 → 0, MAJATNet accuracy improved by 1.5%, 2.49% and 1.67%, respectively. The accuracies of six transfer tasks were improved compared to the CDA method. In the transfer tasks 0 → 2, 1 → 0, and 2 → 0, MAJATNet accuracy improved by 0.76%, 3.24% and 1.91%, respectively, and had a smaller standard deviation. Compared to the domain adaptation methods such as CSAN, LMMD, and JCSDAN, MAJATNet still demonstrates a significant advantage across all tasks. These three domain adaptation methods perform poorly even on relatively easier classification tasks (such as 0 → 1, 0 → 2), which can be attributed to their weak feature extraction modules. Despite employing different domain adaptation techniques, they still fail to learn domain-invariant categorical features. Therefore, optimizing model design from both the perspectives of network architecture and domain alignment is essential to ensure better prediction performance. There are large differences in data distribution across domains, and MAJATNet always obtains better diagnosis performance, which is attributed to the MAResNet structure and the new IJA loss function proposed in this paper. MAJATNet adopts MSGConv to extract multi-scale features and employs an attention mechanism to recalibrate the features across various scales. This approach emphasizes critical scale details while squelching unnecessary scale features. The proposed IJA loss function can gauge the joint distributional differences from multiple perspectives. This is more conducive to the extraction of domain-independent characteristics. Thus, the application of the developed components contributes to a better diagnosis performance; the advantages of the MAJATNet model for UCFD are demonstrated by the results.

(4) **Confusion matrix**

**Figure 11:**
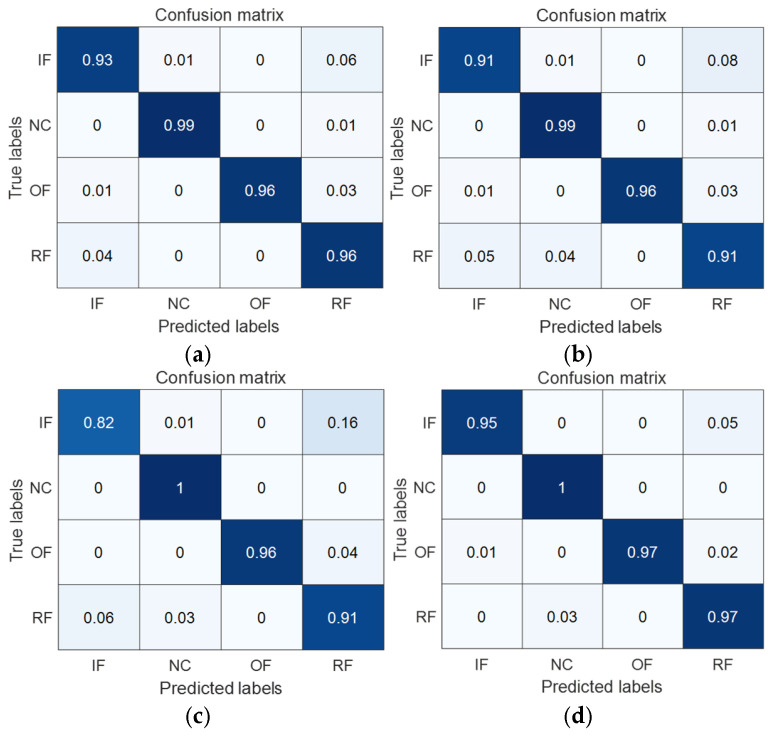
Confusion matrices of different methods in the transfer task 1 → 0. (**a**) MK-MMD; (**b**) DA method; (**c**) CDA method; (**d**) MAJATNet.

**Figure 12:**
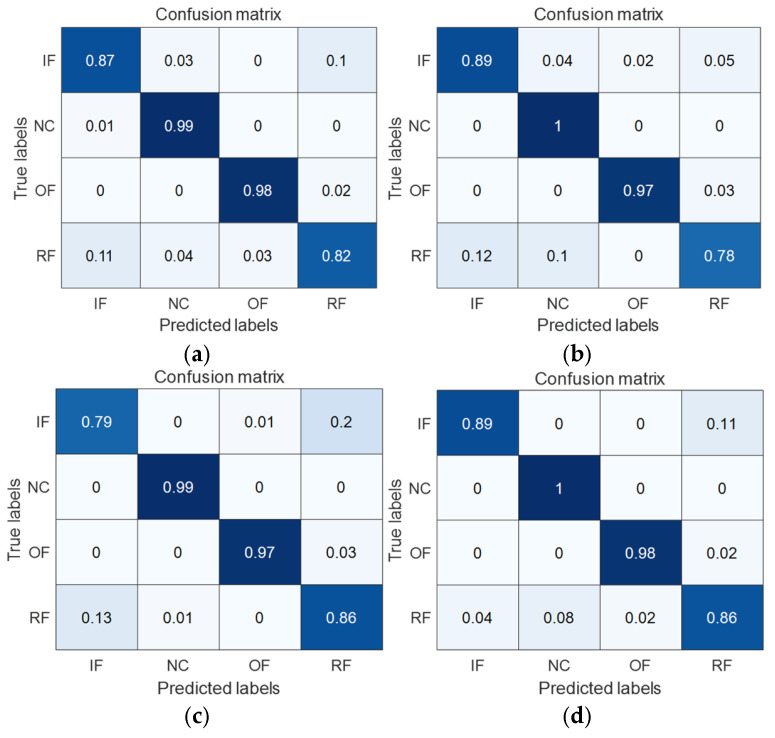
Confusion matrices of different methods in the transfer task 2 → 0. (**a**) MK-MMD; (**b**) DA method; (**c**) CDA method; (**d**) MAJATNet.

To have a clearer view of the model’s classification accuracy on each category, the transfer learning tasks 1 → 0 and 2 → 0, which are more challenging to diagnose by each model, are taken as examples; the confusion matrices of the four methods, MK-MMD, DA, CDA, and MAJATNet, are given in Figure 11 and Figure 12, respectively. Each row in the confusion matrix denotes the predicted label category, and each column denotes the true label category. NC denotes normal condition. IF, OF, and RFRFs denote inner ring, outer ring and rolling element faults, respectively. The data x(i,j) in the confusion matrix denote the ratio of categories in row i that are predicted to be the corresponding category in column j. For example, in Figure 11a, the data in the first row indicates that the true category of inner ring failure is 93% diagnosed as inner ring failure, 1% diagnosed as normal, 0% diagnosed as outer ring failure, and 6% diagnosed as rolling element failure.

As can be seen in Figure 11, in the transfer task 1 → 0, the MAJATNet method achieves more than 95% accuracy on each category. More importantly, the developed model achieves higher accuracy than the MK-MMD, DA and CDA methods in the four categories. A similar phenomenon is observed in the transfer learning task 2 → 0. MAJATNet still obtained the highest diagnosis accuracy in four categories. The confusion matrices results further validate the superiority of the MAJATNet model.

(5) **Feature visualization**

**Figure 13:**
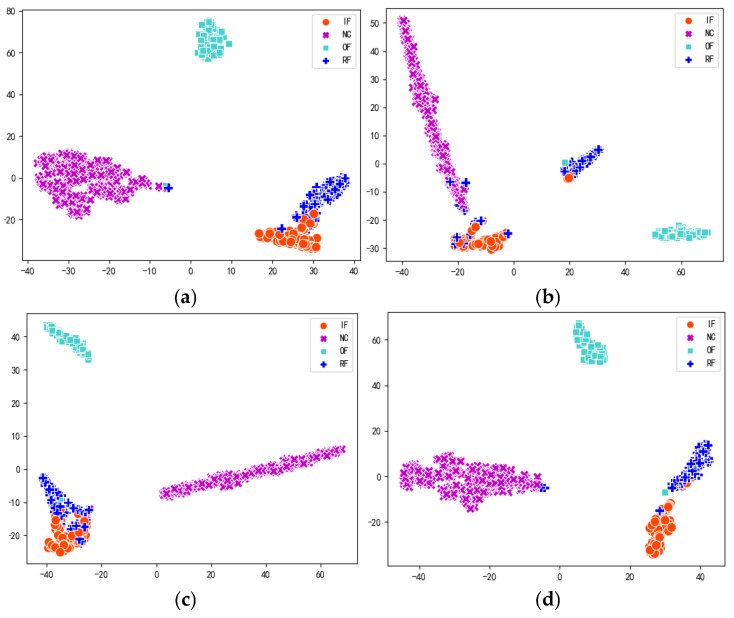
Feature visualization in the transfer task 1 → 0. (**a**) MK-MMD; (**b**) DA method; (**c**) CDA method; (**d**) MAJATNet.

**Figure 14:**
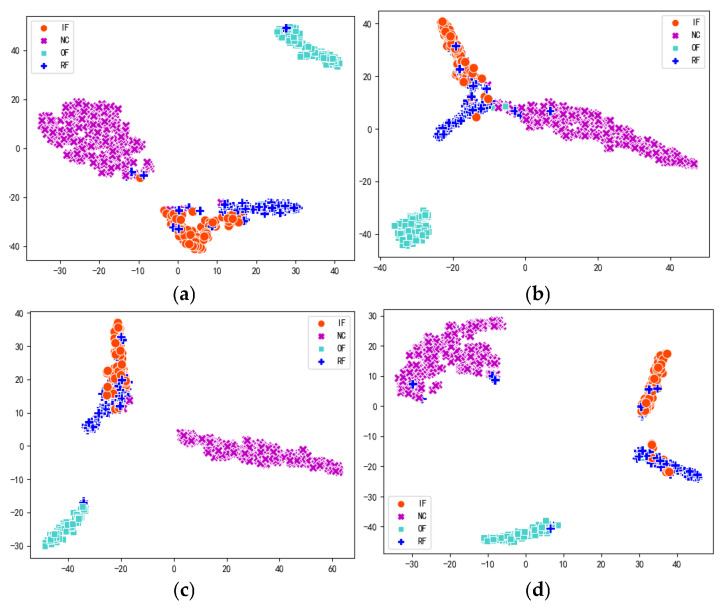
Feature visualization in the transfer task 2 → 0. (**a**) MK-MMD; (**b**) DA method; (**c**) CDA method; (**d**) MAJATNet.

To reveal the model’s feature learning ability, the high-dimensional features are visualized by projecting them into a two-dimensional space. The clustering performance of the different methods in the transfer tasks 1 → 0 and 2 → 0 is shown in Figure 13 and Figure 14. Figure 13 shows that in the transfer task 1 → 0, the inner ring and rolling element fault of MK-MMD are difficult to distinguish from each other, and most of the samples are clustered together. The normal samples of the DA method cannot be clustered together, and there are many rolling element and inner ring failure samples clustered together. The normal samples of the CDA method cannot be clustered together, and a large number of inner ring and rolling element failure samples are mixed together, making it difficult to distinguish. However, MAJATNet ensures that samples from different categories are distinctly separated, while samples within the same category are better clustered together.

In the transfer learning task 2 → 0, MK-MMD and CDA methods show a mix of numerous inner ring and rolling element faults, making them hard to distinguish, while the DA method exhibits a mixture of normal, inner ring fault, and rolling element fault samples. In contrast, MAJATNet has only a small number of inner ring and rolling element failures clustered together. This is due to the ability of the MAJATNet model to reduce the intra-class distance and expand the inter-class distance. The MAJATNet model is more capable of extracting category features and domain-invariant features.

(6) **Visualization of attention weights**

**Figure 15:**
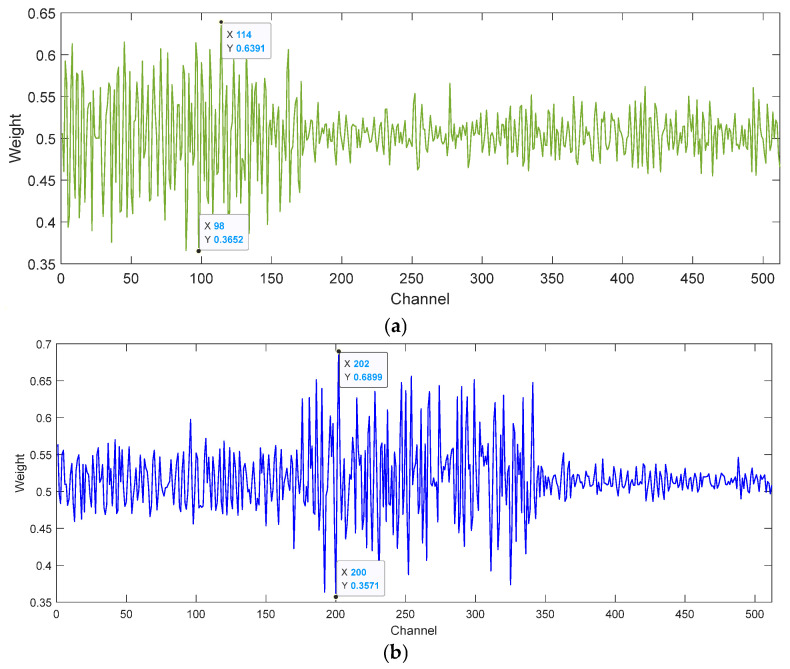
Attention weight visualization results. (**a**) Transfer task 1 → 0; (**b**) Transfer task 2 → 0.

To further confirm that the attention can learn a range of weights for different sizes to emphasize critical scale details while squelching unnecessary scale features, the last attention weights in Figure 5 are visualized. Figure 15a illustrates that in the transfer task 1 → 0, the maximum attention weight is 0.6391, which occurs in the channel 114, highlighting important feature information through larger values. The smallest attention weight is 0.3652, which occurs in channel 98 and suppresses redundant feature information by a value close to zero. As shown in Figure 15b, in the transfer task 2 → 0, the maximum attention weight is 0.6899, which occurs in channel 202. The smallest attention weight is 0.3571, which occurs in channel 200. Attention weight visualization validates the effectiveness of the ECA module.

(7) **Ablation experiments**

MAJATNet adopted a multi-scale group convolution as the domain feature extraction backbone network. An ECA module was employed to recalibrate the scale information of different channels. The IJA loss integrating JMMD and adversarial learning was developed to optimize the model parameters, thereby reducing the differences in domain characteristics. Ablation experiments were conducted to assess the effectiveness of the four components, namely, MSGConv, ECA, JMMD, and adversarial learning in the developed MAJATNet model, which were structured as follows:

Model 1: This model removes the attention mechanism module, JMMD module, and adversarial learning module from the MAJATNet model, and adopts ordinary convolution instead of MSGConv.

Model 2: This model adds the JMMD module to Model 1.

Model 3: Adversarial learning is added to Model 2, forming Model 3.

Model 4: This model adopts MSGConv instead of ordinary convolution in Model 3.

Model 5: This model adds the attention mechanism module to Model 4, which is also the developed MAJATNet model.

Comparing Models 1 and 2 can verify whether the JMMD module is effective; comparing Models 2 and 3 can verify whether the adversarial learning is effective; comparing Models 3 and 4 can verify whether the MSGConv module is effective; comparing Models 4 and 5 can verify whether the attention mechanism is effective. Table 3 presents the ablation experiment results, in which (↑) indicates the current model’s average accuracy increases relative to the previous model, (↓) indicates that it has decreased relative to the previous model, and (-) indicates that it has remained unchanged relative to the previous model.

The average accuracy of Model 2 increases relative to Model 1 in six transfer tasks, especially in task 1 → 0 and task 2 → 0 by 12.83% and 5.77%, respectively. The results indicate the effectiveness of aligning the joint distribution of high-level characteristics and labels between data from two domains via JMMD. The average accuracy of Model 3 increases relative to Model 2 in four transfer tasks (0.31% in 0 → 1, 0.44% in 0 → 2, 0.07% in 1 → 2, 0.14% in 2 → 1) and decreases to a lesser extent in two transfer tasks. The results demonstrate that improving cross-domain fault diagnosis accuracy through adversarial learning is effective in most transfer tasks. The average accuracy of Model 4 increased relative to Model 3 in four transfer tasks (0.47% in 0 → 1, 0.58% in 0 → 2, 0.09% in 1 → 0, and 0.58% in 2 → 0), and remained stable in two transfer tasks. The results indicate that discriminative feature information in the data can be extracted more efficiently by using MSGConv as the backbone network for data from two domains. The average accuracy of Model 5 increased relative to Model 4 in five transfer tasks and decreased to a lesser extent in one transfer task. In particular, accuracy increased by 1.4% and 0.75% in the challenging-to-categorize tasks 1 → 0 and 2 → 0, respectively. The results indicate that the attention mechanism module contributes to recalibrating the importance of feature information at different scales and is effective in improving the diagnosis accuracy of the model.

### 4.2. CWRU Dataset Experimental

(1) **Experimental platform and data processing**

**Figure 16:**
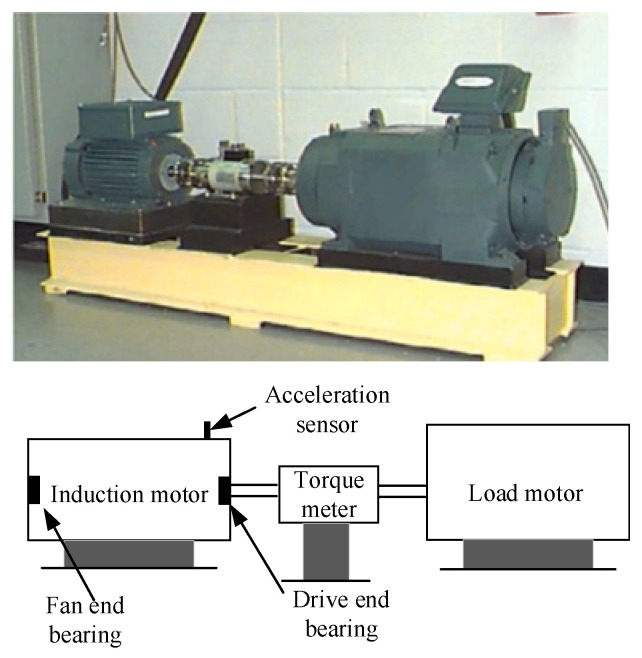
CWRU bearing experimental platform.

As illustrated in Figure 16, the Case Western Reserve University (CWRU) test bed basically includes an induction motor, a torque meter, a load motor, an accelerometer, and bearings. The drive end bearing was selected as the bearing under test and the bearing type was SKF 6205-2RS. The acceleration sensor collects vibration data, and the sampling frequency is 12 kHz. CWRU dataset includes the following: normal, inner ring fault, outer ring fault, and rolling element fault with different-sized cracks. Each failure type contains three different crack sizes of 0.1778 mm, 0.3556 mm, and 0.5334 mm. Therefore, there are a total of 10 categories. The experimental bench was operated at four different load conditions, including 0 HP, 1 HP, 2 HP, and 3 HP, and contained a total of 30 subdatasets. Unlike the JNU bearing dataset, the CWRU bearing dataset includes fault types with varying fault sizes. The number of fault categories increases from 4 in the JNU dataset to 10 in the CWRU dataset, making the diagnosis more challenging. A sample is formed by intercepting 1024 data points in each subdataset. Similarly to the JNU dataset experiments, to evaluate the performance and advantages of the developed MAJATNet model for UCFD, a total of two transfer tasks were designed, which are the more challenging transfer tasks to diagnose in the existing studies [25], as shown in Table 4. 0, 1, 2, and 3 represent bearings serving in four different operating conditions, 0 HP, 1 HP, 2 HP, and 3 HP, respectively, and different operating conditions are used to simulate data from different domains.

(2) **Anti-noise experiment results**

In the CWRU dataset experiments, the maximum training epoch was 100, the pre-trained epoch was 20, and the learning rate decay epochs were 40 and 70. Other hyperparameters remain unchanged. In the actual working scenarios of the bearings, ambient noise from vibration and friction inevitably interferes with the vibration signals, further reducing the accuracy of UCFD. To keep the experimental scenarios more similar to the industrial practical, Gaussian noise signals with signal-to-noise ratios (SNRs) of −2 dB, 0 dB, and 2 dB are inserted into the original signals for the experiments, with the results displayed in Table 5. The results show that all methods’ prediction accuracies degrade as the SNRs decrease. This is due to the addition of Gaussian noise to the original signal, causing the discriminative information to be interfered with by the noise, which increases the challenge of extracting domain-invariant features.

When the SNR is 2 dB, the MAJATNet model can obtain the highest accuracy of 97.93% in the transfer task 2 → 0, which is an improvement of 3.75%, 2.91%, and 6.21% compared to the traditional methods such as CNN, ResNet, and AdaBN, respectively. The accuracy is improved by 3.37%, 3.06%, and 2.68% compared to domain adaptive methods such as MK-MMD, DA, and CDA, respectively. Compared to the four lightweight models—ShuffleResNet, SqueezeNet, LiConvFormer, and MobileNetV2—MAJATNet improves prediction accuracy by 0.84%, 1.84%, 9.58%, and 1.76%, respectively. Notably, it achieves a significant improvement over LiConvFormer, and further research is needed to use the Transformer model for UCFD of bearings. In the transfer task 3 → 0, the MAJATNet model achieves the highest diagnosis accuracy of 98.54%, which is an improvement of 9.41%, 9.57%, 9.39%, 5.36%, 2.98%, and 5.36% compared to CNN, ResNet, AdaBN, MK-MMD, DA, and CDA methods, respectively. Compared to the four lightweight models ShufflResNet, SqueezeNet, LiConvFormer, and MobileNetV2, MAJATNet has improved prediction accuracy by 1.15%, 4.21%, 14.17%, and 3.67%, respectively. The results reveal that the MAJATNet model’s accuracy is significantly improved. The MAJATNet model can maintain the ability to better extract domain-invariant characteristics and domain adaptation under weak noise interference.

When the SNR is decreased to 0 dB, in transfer task 2 0, the MAJATNet model improves the diagnosis accuracy by 5.44%, 5.21%, 7.06%, 4.98%, 3.44%, 3.75%, 2.68%, 4.59%, 13.56%, and 9.11% compared to CNN, ResNet, AdaBN, MK-MMD, DA, CDA, ShufflResNet, SqueezeNet, LiConvFormer, and MobileNetV2 methods, respectively. In the transfer task 3→0, the MAJATNet model improved the accuracy by 11.41%, 9.96%, 8.32%, 5.44%, 2.68%, 6.74%, 1.53%, 5.82%, 16.93%, and 8.88%, respectively. The MAJATNet model has a smaller standard deviation for the five experiments. As the SNR decreases, the MAJATNet model is still able to maintain better anti-noise performance and stability. Particularly in the case of LiConvFormer and MobileNetV2, their prediction accuracy dropped below 90% in two tasks, whereas MAJATNet maintained an accuracy above 97%. Although LiConvFormer and MobileNetV2 achieve a high degree of lightweight design, this comes at the cost of a significant sacrifice in accuracy.

When the SNR decreases to −2 dB, the diagnosis accuracies of CNN, ResNet, AdaBN, MK-MMD, DA, CDA, ShuffeResNet and MobileNetV2 methods in the transfer task 2 → 0 are significantly reduced, and the accuracies can only be maintained at around 90%. SqueezeNet and LiConvFormer can only achieve 80%, whereas the proposed MAJATNet model is able to maintain the maximum accuracy and minimum standard deviation of 94.95% and 0.74%, while the standard deviation of the other methods was much greater than 0.74%. In the transfer task 3 → 0, the accuracy of CNN, ResNet, AdaBN, MK-MMD, DA, CDA, and ShuffeResNet methods significantly decreases and is less than 90%. LiConvFormer, SqueezeNet, and MobileNetV2 are only around 80%, while the MAJATNet model is able to maintain the highest accuracy of 95.02%, with at least 5% improvement. Moreover, the standard deviation of the five experiments was as low as 0.77%, and all other methods were greater than 2%. The MAJATNet model still maintains better anti-noise performance and stability under strong noise interference. The proposed MAJATNet method mainly deals with the impact of noise on diagnostic accuracy through MSGConv and 1D-ECA. Noise usually has specific frequency or scale characteristics, and the multi-scale structure of MSGConv can more comprehensively capture useful information in fault signals while suppressing noise interference. 1D-ECA can automatically identify and enhance fault-sensitive feature channels and suppress noise-dominated channels, which contributes to the application of the proposed method to real industrial environments.

## 5. Conclusions

Considering the challenging multi-scale feature extraction, the calculation of marginal distribution only, and the insufficiency of a single loss function to accurately measure the distributional discrepancy in existing methods for the UCFD of bearings, this paper proposes a new lightweight-based multi-scale attention joint adaptive adversarial transfer network, namely MAJATNet. The developed model includes feature extraction network innovation and loss function improvement. The MAResNet module was designed for extracting features from data of two domains; the proposed IJA loss function updates the model parameters during the training phase. Extensive experiments have been conducted on the JNU and CWRU bearing datasets, and the developed model overcomes the existing limitations and improves the fault diagnosis accuracy. The results confirm that the developed model has the best diagnosis performance and has obvious advantages for UCFD. In future research, we plan to apply the MAJATNet model to more challenging UCFD scenarios involving bearings with multi-source domains and label inconsistencies to further enhance the practicality of the developed approach.

## Figures and Tables

**Table 1:** Hyperparameter settings.

Hyperparameters	Parameters Information
Batch size	64
Optimization algorithm	Adam
Momentum	0.9
Epoch	300
Learning rate	0.001
Learning rate strategy	Step
Learning rate decay coefficient	0.1
Learning rate decay epoch	150, 250
Weight decay coefficient of the L2	0.00001

**Table 2:** Fault diagnosis accuracy in different tasks.

Method	Complexity	Parameters $\mathrm{of}\;G_{f}$	Inference Time	0 → 1	0 → 2	1 → 0	1 → 2	2 → 0	2 → 1
CNN	18.23 MMac	181.25 K	0.04 s	98.33 ± 0.52	96.21 ± 0.60	82.97 ± 1.52	98.87 ± 0.31	90.34 ± 0.95	98.39 ± 0.26
ResNet	986 MMac	3.84 M	0.32 s	98.60 ± 0.53	96.56 ± 0.25	83.93 ± 3.85	99.01 ± 0.08	87.95 ± 1.58	99.39 ± 0.33
AdaBN	986 MMac	3.98 M	14.6 s	94.62 ± 0.45	93.04 ± 0.95	88.47 ± 1.17	94.61 ± 0.30	90.30 ± 0.89	95.77 ± 0.58
MK-MMD	986 MMac	3.84 M	0.28 s	99.18 ± 0.25	98.53 ± 0.23	96.93 ± 0.12	99.80 ± 0.08	93.59 ± 0.31	99.63 ± 0.30
DA	986 MMac	3.84 M	0.28 s	99.05 ± 0.49	98.02 ± 0.50	95.46 ± 0.35	99.59 ± 0.15	93.31 ± 0.76	99.42 ± 0.09
CDA	986 MMac	3.84 M	0.28 s	99.22 ± 0.19	98.76 ± 0.32	94.71 ± 0.47	99.66 ± 0.12	93.07 ± 0.41	99.56 ± 0.09
ShuffeResNet	51 MMac	1.1 M	0.08 s	99.69 ± 0.22	99.22 ± 0.26	96.18 ± 0.60	99.83 ± 0.00	93.86 ± 0.17	99.90 ± 0.09
SqueezeNet	94 MMac	359 K	0.10 s	98.67 ± 0.44	98.50 ± 0.33	93.31 ± 0.28	99.56 ± 0.26	92.35 ± 0.46	99.73 ± 0.15
LiConvFormer	14 MMac	320 K	0.06 s	98.16 ± 0.48	97.30 ± 0.69	93.79 ± 0.77	98.33 ± 0.56	92.69 ± 0.57	98.09 ± 0.48
MobileNetV2	96 MMac	2.18 M	0.11 s	98.50 ± 0.48	97.27 ± 1.05	93.28 ± 0.46	99.32 ± 0.12	92.52 ± 0.35	99.35 ± 0.19
CSAN	-	-	-	94.34	92.59	91.45	90.55	87.53	93.78
LMMD	-	-	-	98.12	94.17	93.80	95.94	88.54	97.50
JCSDAN	-	-	-	98.54	95.00	94.69	97.08	89.12	97.95
MAJATNet	569 MMac	2.21 M	0.28 s	**99.90 ± 0.09**	**99.52 ± 0.22**	**97.95 ± 0.42**	**99.86 ± 0.08**	**94.98 ± 0.26**	**99.90 ± 0.09**

**Table 3:** Results of ablation experiments.

Transfer Task	Model 1	Model 2	Model 3	Model 4	Model 5
0 → 1	98.60 ± 0.53	99.08 ± 0.26 (↑)	99.39 ± 0.26 (↑)	99.86 ± 0.22 (↑)	**99.90 ± 0.09** (↑)
0 → 2	96.56 ± 0.25	98.57 ± 0.36 (↑)	99.01 ± 0.52 (↑)	99.59 ± 0.23 (↑)	**99.52 ± 0.22** (↓)
1 → 0	83.93 ± 3.85	96.76 ± 0.56 (↑)	95.56 ± 0.49 (↓)	96.55 ± 0.74 (↑)	**97.95 ± 0.42** (↑)
1 → 2	99.01 ± 0.08	99.73 ± 0.09 (↑)	99.80 ± 0.08 (↑)	99.80 ± 0.08 (-)	**99.86 ± 0.08** (↑)
2 → 0	87.95 ± 1.58	93.72 ± 0.28 (↑)	93.65 ± 0.39 (↓)	94.23 ± 0.56 (↑)	**94.98 ± 0.26** (↑)
2 → 1	99.39 ± 0.33	99.66 ± 0.12 (↑)	99.80 ± 0.08 (↑)	99.80 ± 0.14 (-)	**99.90 ± 0.09** (↑)

**Table 4:** CWRU dataset transfer tasks.

Transfer Task	Source Domain	Target Domain	Number of Samples in Source Domain	Number of Samples in Target Domain
2 → 0	2 HP	0 HP	1539	1305
3 → 0	3 HP	0 HP	1544	1305

**Table 5:** CWRU dataset experimental results.

Transfer Task	2 → 0	3 → 0	2 → 0	3 → 0	2 → 0	3 → 0
SNR	2 dB	2 dB	0 dB	0 dB	−2 dB	−2 dB
CNN	94.18 ± 1.22	89.13 ± 4.31	92.64 ± 0.84	85.75 ± 2.48	88.04 ± 0.83	84.14 ± 3.21
ResNet	95.02 ± 1.43	88.97 ± 2.48	92.87 ± 1.55	87.20 ± 1.45	90.27 ± 2.11	83.93 ± 2.16
AdaBN	91.72 ± 0.49	89.15 ± 1.43	91.02 ± 1.32	88.84 ± 1.30	86.82 ± 1.18	81.98 ± 2.52
MK-MMD	94.56 ± 0.50	93.18 ± 2.07	93.10 ± 1.24	91.72 ± 2.31	90.65 ± 1.64	89.89 ± 2.15
DA	94.87 ± 0.92	95.56 ± 0.84	94.64 ± 1.09	94.48 ± 0.96	91.42 ± 1.68	88.04 ± 2.86
CDA	95.25 ± 0.93	93.18 ± 2.29	94.33 ± 1.37	90.42 ± 2.53	91.19 ± 1.35	89.04 ± 2.81
ShuffeResNet	97.09 ± 1.17	97.39 ± 0.78	95.40 ± 0.90	95.63 ± 0.96	92.72 ± 1.21	90.73 ± 0.95
SqueezeNet	96.09 ± 0.42	94.33 ± 1.52	93.49 ± 1.97	91.34 ± 2.34	81.07 ± 6.33	77.04 ± 3.89
LiConvFormer	88.35 ± 1.11	84.37 ± 4.84	84.52 ± 2.24	80.23 ± 3.03	78.24 ± 4.43	72.03 ± 3.76
MobileNetV2	96.17 ± 1.08	94.87 ± 2.73	88.97 ± 2.95	88.28 ± 4.70	88.20 ± 1.44	83.91 ± 6.27
MAJATNet	**97.93 ± 0.88**	**98.54 ± 0.42**	**98.08 ± 0.61**	**97.16 ± 0.64**	**94.95 ± 0.74**	**95.02 ± 0.77**

## Data Availability

The data presented in this study are available on request from the corresponding author.
